# Correlation of HIV-Induced Neuroinflammation and Synaptopathy with Impairment of Learning and Memory in Mice with HAND

**DOI:** 10.3390/jcm12165169

**Published:** 2023-08-08

**Authors:** Kaspar Keledjian, Tapas Makar, Chenyu Zhang, Jiantao Zhang, Bosung Shim, Harry Davis, Joseph Bryant, Volodymyr Gerzanich, J. Marc Simard, Richard Y. Zhao

**Affiliations:** 1 Department of Neurosurgery, University of Maryland School of Medicine, Baltimore, MD 21201, USA; kkeledjian@som.umaryland.edu (K.K.); tapasmakar@hotmail.com (T.M.); bosung.shim@som.umaryland.edu (B.S.); vgerzanich@som.umaryland.edu (V.G.); 2 Department of Pathology, University of Maryland School of Medicine, Baltimore, MD 21201, USA; chenyu.zhang@som.umaryland.edu (C.Z.); jiantao.zhang@som.umaryland.edu (J.Z.); 3 Institute of Human Virology, University of Maryland School of Medicine, Baltimore, MD 21201, USA; hdavis@ihv.umaryland.edu (H.D.); jbryant@ihv.umaryland.edu (J.B.); 4 Surgical Care Clinical Center, VA Maryland Health Care System, Baltimore, MD 21201, USA; 5 Department of Microbiology-Immunology, University of Maryland School of Medicine, Baltimore, MD 21201, USA; 6 Institute of Global Health, University of Maryland School of Medicine, Baltimore, MD 21201, USA; 7 Research & Development Service, VA Maryland Health Care System, Baltimore, MD 21201, USA

**Keywords:** HIV/AIDS, HIV-associated neurocognitive disorders (HAND), neuroinflammation, synaptopathy, transgenic HIV mice, SUR1-TRPM4, learning and memory

## Abstract

Over 38 million people worldwide are living with HIV/AIDS, and more than half of them are affected by HIV-associated neurocognitive disorders (HAND). Such disorders are characterized by chronic neuroinflammation, neurotoxicity, and central nervous system deterioration, which lead to short- or long-term memory loss, cognitive impairment, and motor skill deficits that may show gender disparities. However, the underlying mechanisms remain unclear. Our previous study suggested that HIV-1 infection and viral protein R (Vpr) upregulate the SUR1-TRPM4 channel associated with neuroinflammation, which may contribute to HAND. The present study aimed to explore this relationship in a mouse model of HAND. This study employed the HIV transgenic Tg26 mouse model, comparing Tg26 mice with wildtype mice in various cognitive behavioral and memory tests, including locomotor activity tests, recognition memory tests, and spatial learning and memory tests. The study found that Tg26 mice exhibited impaired cognitive skills and reduced learning abilities compared to wildtype mice, particularly in spatial memory. Interestingly, male Tg26 mice displayed significant differences in spatial memory losses (*p* < 0.001), while no significant differences were identified in female mice. Consistent with our early results, SUR1-TRPM4 channels were upregulated in Tg26 mice along with glial fibrillary acidic protein (GFAP) and aquaporin 4 (AQP4), consistent with reactive astrocytosis and neuroinflammation. Corresponding reductions in neurosynaptic responses, as indicated by downregulation of Synapsin-1 (SYN1) and Synaptophysin (SYP), suggested synaptopathy as a possible mechanism underlying cognitive and motor skill deficits. In conclusion, our study suggests a possible relationship between SUR1-TRPM4-mediated neuroinflammation and synaptopathy with impairments of learning and memory in mice with HAND. These findings could help to develop new therapeutic strategies for individuals living with HAND.

## 1. Introduction

There are approximately 38 million people currently living with human immunodeficiency virus (HIV) and acquired immunodeficiency syndrome (AIDS) globally. Antiretroviral therapies (ARTs) can effectively eliminate active replicating viruses, prolonging the lives of infected individuals. However, a significant challenge arises when certain HIV drugs poorly penetrate the central nervous system (CNS) due to the presence of the blood–brain barrier. As a result, despite achieving viral suppression in the blood, the virus can persistently replicate and cause inflammation in the brain. This chronic inflammation and ongoing viral activity contribute to the deterioration of the CNS and lead to the development of HIV-associated neurocognitive disorders (HAND) in patients [1,2]. More than half of those infected are afflicted with HAND, with significant impact on aging individuals [3,4,5]. Although ART can significantly decrease the severe and progressive deterioration of mental functions, long-term brain inflammation often persists, resulting in high rates of delirium, dementia, and depression, which can lead to suicide. The risk of suicide in HIV-infected persons is 3–5 times higher than in their HIV-uninfected counterparts [6].

HIV-1 infection in the brain often causes profound inflammation through inciting NF-kB-mediated proinflammatory cytokines such as TNFα [7,8], leading to neuronal cell and brain damage that results in HAND and prevents proper brain functions [3,9]. Consequently, individuals with HAND are often afflicted with short- or long-term memory loss and impaired motor abilities. Gender disparities have also been reported in these deficiencies [10,11,12,13]. For example, compared with HIV-infected women, HIV-infected men have shown poorer performance in verbal memory tests, whereas cocaine-dependent HIV-infected women have shown poorer episodic memory [11,12,13]. Although it is known that HIV-associated long-lasting decreased mental functions are typically characterized by brain inflammation and brain cell death, how HIV causes decreased mental functions over an extended period remains poorly understood.

Neurocognitive and neurodegenerative changes resulting from HIV-1 infection have been replicated in rodent models [14,15,16]. An HIV-1 transgenic 26 (Tg26) mouse model has been established [17] to explore the long-term effects of HIV infection and HAND [7,18]. In Tg26 mice, HIV-1 infection leads to neuroinflammation, astrogliosis, and neuronal cell damage [7,18]. Furthermore, expression of HIV-1 genes in the hippocampal dentate gyrus of Tg26 mice consistently shows reduced hippocampal neurogenesis and dendritic abnormalities in new-born mature granule neurons [18], suggesting a direct impact of HIV-1 infection on hippocampal function. Specifically, transgenic mice that specifically produce the viral Tat (transactivator of transcription) protein or the viral protein R (Vpr) protein in the hippocampus show synaptic loss that is associated with learning and memory deficits [19,20]. However, the onset of cognitive impairments and the underlying synaptic mechanisms in Tg26 mice have not been investigated. As with HIV-infected humans, sex differences in response to HIV-1 infection are also observed in HAND animal models [21,22,23]. However, the underlying mechanisms remain largely unknown. Therefore, understanding the role of biological sex in HAND and its impact on cognitive impairments is important, as it may reveal pathways that can be targeted to optimize HAND treatments.

Our studies have extended beyond early investigations to demonstrate a unique functional relationship between HIV-1 infection and the upregulation of the SUR1-TRPM4 channel, which contributes to neuroinflammation and may be implicated in HAND and cognitive impairment [7]. The SUR1-TRPM4 channel, which consists of SUR1 (sulfonylurea receptor 1) and TRPM4 (transient receptor potential melastatin 4) heteromers, regulates Ca^2+^ influx [24,25]. It is not constitutively expressed, but is transcriptionally upregulated in microglia and astrocytes in response to various neuroinflammatory brain conditions, including traumatic brain injury (TBI), subarachnoid hemorrhage (SAH), and neuroinflammatory diseases such as multiple sclerosis (MS)/experimental autoimmune encephalomyelitis (EAE) [25,26,27,28]. We have also demonstrated that the SUR1-TRPM4 channel is elevated in astrocytes of HIV-infected postmortem human brain tissues and in the hippocampi of Tg26 mice [7]. Furthermore, we showed that the expression of HIV-1 Vpr in astrocytic SNB19 cell lines elicits the same proinflammatory biomarkers as the SUR1 protein [7]. Earlier studies demonstrated that astrocytic expression of HIV-1 Vpr causes neuroinflammation [29] and synaptic loss and memory impairment [20]. Thus, transcriptional activation of the SUR1-TRPM4 channel by HIV-1 infection or Vpr may play a key role in regulating not only brain injuries but also HAND [25,30,31]. However, it remains unknown whether there is a direct connection between SUR1-mediated neuroinflammation and synaptic loss and memory impairment.

The aim of this study was to investigate the relationship between SUR1-TRPM4-mediated neuroinflammation and learning and memory in Tg26 mice. Tg26 transgenic mice were selected for their similarity to HAND pathologies and their relevance to HIV-1 and viral protein-induced neuroinflammation [7,17,18]. We previously demonstrated that HIV-1 infection induces SUR1-mediated neuroinflammation and neurotoxicity in Tg26 mice [7]. In this study, we conducted a range of neurobehavioral and cognitive tests to evaluate learning and memory abilities and motor mobility in Tg26 mice and compared these behaviors with those of wildtype (WT) mice, both male and female. We measured possible molecular changes in the hippocampus and cortex regions of the brain using immunohistochemistry (IHC) and quantitative reverse transcription–polymerase chain reaction (qRT-PCR) analyses to determine the expression of the SUR1-TRPM4 channel, neuroinflammatory markers for reactive astrocytosis (glial fibrillary acidic protein (GFAP) and aquaporin 4 (AQP4)), and markers for synapses (synaptophysin (SYP) and synapin-1 (SYN1)).

GFAP is a well-established biomarker for reactive astrocytosis due to its astrocyte-specific expression and upregulation in response to brain injury. Astrocytes become activated when the brain is damaged or insulted, resulting in increased GFAP expression. Therefore, detecting GFAP through immunostaining and other techniques is essential for investigating the cellular response to brain injury and disease [32]. GFAP elevation in reactive astrocytes is also associated with HIV-1 infection [33], and transcriptional activation of the SUR1-TRPM4 channel that regulates proinflammatory responses [26,31]. AQP4 is a water channel protein that is primarily expressed in astrocytes in the brain and plays a critical role in regulating water transport across the blood–brain barrier and within the CNS. AQP4 has also been found to be upregulated in reactive astrocytes in various CNS pathologies, such as TBI, stroke, MS, and neuromyelitis optica [34]. Similar to GFAP, AQP4 has been suggested as a potential marker for reactive astrogliosis since it is selectively upregulated in astrocytes in response to CNS injury and disease [35].

We also measured SYN1 and SYP here because they are both widely used as markers of neuronal synapses. SYN1 is a phosphoprotein that is found in presynaptic nerve terminals, where it plays a role in regulating synaptic vesicle trafficking and neurotransmitter release. SYP, on the other hand, is a membrane glycoprotein that is abundant in presynaptic vesicles and plays a critical role in vesicle exocytosis and recycling. Both SYN1 and SYP have been used in studies of cognitive behavior and memory [36].

We compared the synaptopathy observed in the Tg26 mice with the WT mice and correlated these changes with molecular alterations in the hippocampus and cortex regions of the mouse brains. We also investigated possible sex differences in the observed synaptopathy and cognitive impairments.

## 2. Materials and Methods

### 2.1. Ethics Statement

The University of Maryland, Baltimore, Institutional Animal Care and Use Committee (IACUC) approved all animal-related experiments (Protocol No.: 1119012, titled “Viral Protein R (Vpr) in HIV-associated Brain Neuroinflammation and Neurotoxicity”), in accordance with guidelines of the National Institute of Health. The study was conducted in compliance with the ARRIVE (Animal Research: Reporting of In Vivo Experiments) guidelines. We certify that all applicable institutional and governmental regulations concerning the ethical use of animals were followed during the course of this research. All efforts were made to minimize the number of animals used and their suffering.

### 2.2. Animals

HIV-1 transgenic Tg26 mice FVB/N expressing high levels of 7 of the 9 HIV-1 proteins were established using the 7.4 kb transgene construct lacking the 3 kb sequence overlapping the *Gag*/*Pol* region of provirus pNL4-3 as described previously [37]. The model was originally obtained from the National Institute of Dental Research, and the colony has been maintained in the Institute of Human Virology (IHV) since 1995 by cross breeding heterozygous mice and continuously backcrossing to the wildtype FVB/N. To ensure little to no genetic drift, we also obtained colonies from Jackson Labs for comparison. WT mice with an FVB/N genetic background generated from the same litter of Tg26 mice were used as controls for these studies. Both WT and Tg26 mice in the age of 12–14 weeks were used for the behavioral tests, and at 16 weeks for the immunostaining and qRT-PCR studies. Tg26 mice were housed under pathogen-free conditions at the animal facility of the IHV, University of Maryland School of Medicine, Baltimore.

### 2.3. Behavior

#### 2.3.1. The Open Field (OF) Test

The OF test is used to determine the gross locomotor ability of mice. The mice were placed in an open field made of clear plexiglass box (16 × 16 × 40 inch) and equipped with a video tracking system (Any-Maze, Stoelting Co, Wood Dale, IL, USA) as described previously [38]. Each mouse was placed in the middle of the open field for a single 5 min (min) session with the experimenter out of view. The homogeneously illuminated floor space was divided into equal squares, corners, center, and intermediary regions. The measurements recorded were time spent in center, time spent in 4 corners, average speed, and distance traveled. After each trial, fecal boli were removed and the floor was wiped clean with 70% alcohol and dried.

#### 2.3.2. The Novel Object Recognition (NOR) Test

The NOR test is used to evaluate short-term memory in rodents with CNS disorders. It is a relatively fast and efficient means for testing different phases of learning and memory in mice. The NOR test was performed as described [39] and consisted of pretraining, training, and testing. The pretraining phase was accomplished during the OF test. All mice were habituated to the open field apparatus (16 × 16 × 14 inch) without any object for 5 min. The following day, mice were trained in the same field with two similar objects for 5 min. The objects used were of similar size, made of easy-to-clean plastic materials. During the acquisition phase, two identical objects were set in the field at a distance from each other. The animal placed in the testing arena was allowed to explore freely for 5 min and was then returned to its home cage. The objects were replaced by another set of objects, one identical and one novel; after 1 h, the animal was returned to the arena and allowed to explore the objects for 5 min (the recognition phase). The time spent interacting with each object as well as the overall time exploring the objects, whether old or new (total exploration time), was measured. The recognition index was calculated as follows: time spent interacting with the novel object/total exploration time × 100. Animals that are able to discriminate the new from the old object should have a recognition index/exploratory preference greater than 50%.

#### 2.3.3. The Novel Object Location (NOL) Test

The NOL test evaluates spatial memory and discrimination abilities of mice with CNS disorders. Mice were tested 2 days after the NOR test (See Section 3.1.2. for the Design). To carry out this experiment, a spatial memory task was used, in which mice were first allowed to explore three objects in an open field box which was the same as the OF arena (See Section 3.1.2. for the Design). One hour later, they were retested to determine their ability to recognize a particular object that had been moved (See Section 3.1.2. for the Design), hence testing short-term memory. Specifically, the objects used were equal in shape and size, and they were plastic objects with no natural significance to mice. Mice were permitted to explore three objects placed in a diagonal arrangement for a 5 min acquisition trial. Mice were removed, and the arena and objects were thoroughly cleaned with 70% alcohol. One hour later, memory retention was probed by returning the mouse to the arena in which one object had been moved to a new location and giving the mouse 5 min to explore the objects. The amount of time each animal spent exploring the objects was calculated and is presented as % time spent with novel object/location over the sum of time spent with all objects. Object exploration was defined as sniffing or touching an object with the snout at a distance of <1 cm from the object [40,41].

#### 2.3.4. The Barnes Maze (BM) Test

The BM test measures a mouse’s ability in spatial learning and memory. A commercially available Barnes maze with 20 holes around the periphery was used (Stoelting Co., Wood Dale, IL, USA). The maze itself was isolated from the rest of the room with black curtains to exclude any external cues which could potentially interfere with the results [42]. Different spatial cues, consisting of simple geometric figures (square, circle, triangle), were hung on the curtain walls. Before the 4 days of acquisition training, a habituation phase took place, during which the mouse was placed on the maze and given 1 min to find the escape hole. If the mouse failed, the experimenter gently guided the mouse to the escape hole and sequestered it there for 1 min. The acquisition trials were performed after the habituation phase, while the maze was cleaned with 70% alcohol. These trials were performed 4 times per day with each mouse [43], with an inter-trial interval of 30 min [42]. On day 5, the short-term memory retention test was performed. During this time, the escape tunnel was replaced with a shallow tray to close the escape tunnel so that it was similar to the other closed holes, and the mouse explored the maze for 2 min. Time spent in the quadrant of the blocked escape tunnel was calculated.

### 2.4. Immunostaining of Brain Tissues

The IHC staining and brain tissue processing have been described previously [7]. Briefly, the brains of both Tg26 and WT control mice were fixed in formalin and subsequently embedded in paraffin. Microtome sectioning was performed on paraffin-embedded mouse brain blocks containing the hippocampus. These sections, 10 μm thick, were subjected to IHC staining using VECTASTAIN ABC kits (Vector Laboratories, Burlingame, CA, USA). The sections were deparaffinized and rehydrated by passing through graded ethanol (100%, 95%, and 70%) and xylene water washes. After antigen retrieval, the samples were preincubated in blocking solution [10% fetal bovine serum (FBS)] in Tris-buffered saline and 0.2% Tween-20 (TBST) for one hour. Subsequently, the samples were incubated overnight at 4 °C in primary antibody-containing blocking solution. Species–specific secondary antibody-containing solutions were then used for a 45 min incubation, followed by avidin–biotin complex (ABC) for 30 min, and finally, in 3,3′-diaminobenzidyne (DAB) solution until a robust color change was observed. Hematoxylin was used to counterstain the nuclei. The antibodies and dilutions used in this study are listed in Table S1.

### 2.5. Histological Image Analysis

To ensure unbiased analysis, the investigator was blinded to the transgenic status of the mice during image analysis. Immunostained brain sections were observed under a light microscope at 40× magnification. Raw images were obtained from 4–6 hippocampal sections per mouse brain and analyzed using ImageJ v1.54 (Fiji) software. DAB substrate-positive cells or regions were identified and counted or measured as integrated density, respectively.

### 2.6. Quantitative Reverse Transcription PCR

The qRT-PCR protocol used in this study was described by Li et al. [7] with modification. Briefly, snap-frozen mouse brain tissues of WT or Tg26 mice at 16 weeks of age with mixed sexes were homogenized using a Precellys Evolution Tissue Homogenizer (Bertin Technologies (Montigny-le-Bretonneux, France)). TRIzol (ThermoFisher (Waltham, MA USA)) was used to extract total RNA from the homogenized tissues, following the manufacturer’s protocol. DNase-treated total RNA (1 µg) was reverse transcribed to cDNA using the MultiScribe Reverse Transcriptase Kit (ThermoFisher) in accordance with the manufacturer’s instructions. The RNA reverse transcription reaction was carried out at 25 °C for 10 min, followed by 37 °C for 2 h and 85 °C for 5 min. Gene amplification was conducted using the PowerTrack SYBR Green Master Mix Kit (ThermoFisher) and a QuantStudio 3 system (ThermoFisher) with the following conditions: 95 °C for 1 min; 40 cycles of 95 °C for 15 s; and 60 °C for 60 s. A melting curve was included for verification of specific gene amplification. The 2^−ΔCT^ value was calculated to determine the relative ratio of HIV-1 viral gene to a house-keeping glyceraldehyde-3-phosphate dehydrogenase (*Gapdh*) gene in each sample. Additionally, the 2^−ΔΔCT^ value was calculated to determine the fold-change in respective mRNA expression in Tg26 relative to WT mouse brains, with *Gapdh* mRNA serving as an endogenous control. The primer sequences that target the genes of interest are provided in Table S2.

### 2.7. Statistical Analysis

Statistical analyses were conducted using GraphPad Prism 8 software (San Diego, CA, USA), and unpaired *t*-tests with Welch correction were utilized to determine statistical significance of the cognitive behaviors and memory, IHC staining, and qRT-PCR analysis of cortex and hippocampus brain tissues between the WT and Tg26 mice groups. The Welch correction was used due to the differences in the variations of the WT and Tg26 groups, as well as the unequal sample sizes of the tested mice. The level of statistical significance was set at 95% confidence levels with *, *p* < 0.05; **, *p* < 0.01; ***, *p* < 0.001.

## 3. Results

### 3.1. Comparison of Tg26′s Motor Mobility, Learning and Memory with WT Mice

The goal of this experiment was to assess and compare the motor mobility, learning, and memory abilities of Tg26 transgenic mice with those of WT control mice. As described below, a range of neurobehavioral and cognitive tests were performed to evaluate the performance of both groups of mice in these tests. The behavioral changes in the Tg26 mice were further compared with those of WT mice, and the results were analyzed by statistics to determine any possible statistically significant differences between the two groups. Possible sexual differences in the observed behavioral and cognitive changes were also analyzed and presented. This study was carried out under an approved IACUC protocol (No.: 1119012), by the University of Maryland, Baltimore in accordance with guidelines of the National Institute of Health.

#### 3.1.1. Test of Locomotor Activity and Recognition Memory

**Figure 1:**
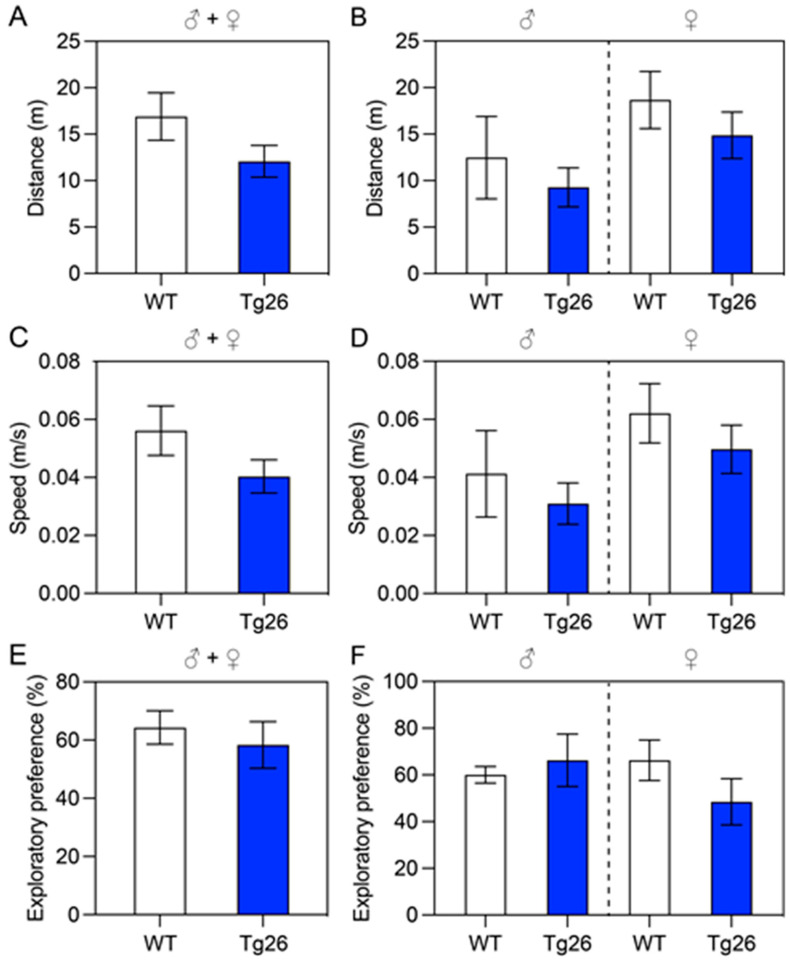
Evaluation of locomotor activity and recognition memory of Tg26 and WT mice. Mice at the age of 12–14 weeks old were used in these experiments. Locomotor activity was assessed using the OF test (**A**–**D**); cognition-related recognition memory was evaluated using the NOR test (**E**,**F**). (**A**) The total distances traveled by Tg26 (*n* = 20) and WT (*n* = 14) mice in the open field test over a period of 5 min, with male (♂) and female (♀) mice shown. (**B**) Sex differences between the Tg26 and WT male (**left**) or female (**right**) groups. The same groups of mice were used to determine differences in average speed of combined male and female groups (**C**) or male or female separately (**D**). The Novel Object Recognition Index/exploratory preference was calculated as described in the Materials and Methods section, with differences between Tg26 and WT mice presented in (**E**) for the combined sex groups and in subgroups (**F**). All bar graphs are shown as Mean ± SEM.

We first evaluated locomotor activity and recognition memory of the Tg26 and WT mice. The OF test was used to quantify the locomotor activity (Figure 1A–D), and the NOR test was used to evaluate cognition, particularly recognition memory in the Tg26 and WT mice (Figure 1E,F). In the OF test, the total distances traveled by the Tg26 and WT mice were compared for both male (♂) and female (♀) groups (Figure 1A) during the 5 min test time period, and possible sex differences in the male and female groups of Tg26 and WT mice (Figure 1B). In addition, the average speed of Tg26 and WT mice during the 5 min test time period in the combined male and female groups or male or female separately are also shown in Figure 1E,F, respectively. Overall, there is a general trend that the Tg26 mice performed poorer than the WT controls. The locomotor activity as measured by the OF test showed that the total distance traveled by the Tg26 mice was shorter (12.08 ± 7.65 vs. 16.90 ± 9.57 m) than the WT mice (Figure 1A) with slower (0.04 ± 0.03 vs. 0.06 ± 0.03 m/s) average speed (Figure 1C), and similar differences were also observed in both the male group (Figure 1B, left) and the female group (Figure 1B, right). However, based on the statistical analysis, those observed differences were not significant. A similar trend was also seen in the NOR test in which the Tg26 mice had lower exploratory preference compared to the WT mice (58.37 ± 33.91 vs. 64.33 ± 22.86%) (Figure 1E). Overall, the results suggest that Tg26 mice displayed a trend toward impaired locomotor activity and recognition memory compared to WT mice.

#### 3.1.2. Test of Spatial Memory and Discrimination Ability

**Figure 2:**
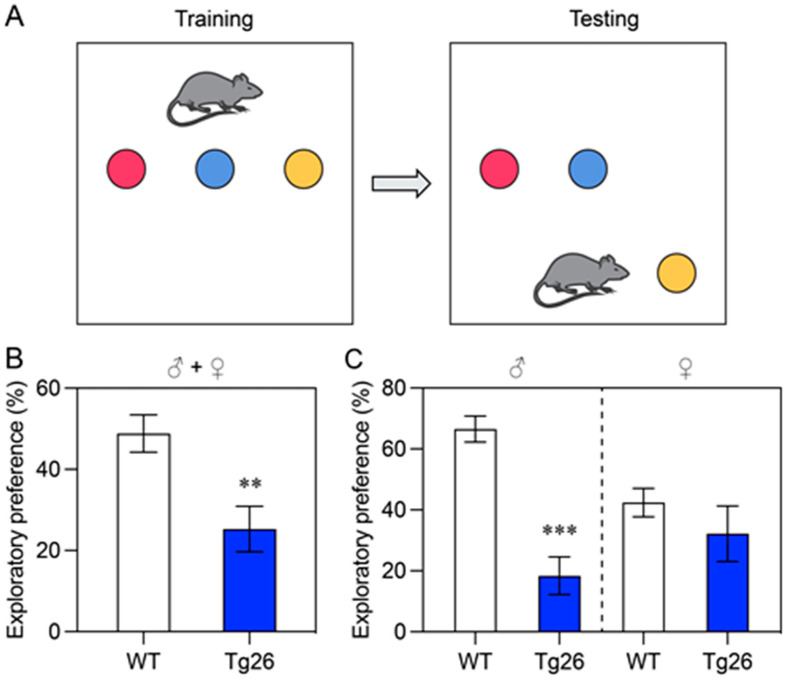
Evaluation of the spatial memory and discrimination abilities of Tg26 and WT mice in the age of 12–14-week-olds using the NOL memory task. (**A**) Schematic representation of the NOL task, which includes the training session (**left**) and the test phase (**right**). During the training session, mice freely explore three different objects, while during the test phase, a familiar object (yellow) is placed in a novel location. (**B**) Results of NOL memory measured in Tg26 and WT mice. The exploratory preference is presented as a percentage of time that male (♂) and female (♀) Tg26 (*n* = 15) and WT (*n* = 20) mice spent exploring the correct object. The retention probe was performed 1 h after the initial acquisition trial. Sex differences within the Tg26 and WT male or female groups are presented in (**C**). All bar graphs are shown as Mean ± SEM, with statistical significance indicated as ** (*p* < 0.01) or *** (*p* < 0.001).

Next, we evaluated the spatial memory and discrimination abilities of Tg26 mice and WT mice using the NOL memory test. The mice were allowed to explore three objects in an open field box, and then were retested one hour later to determine their ability to recognize a relocated (yellow) object and test their short-term memory (Figure 2A). Tg26 mice displayed significantly poorer short-term and spatial memories compared to WT mice (*p* < 0.01) (Figure 2B). Even though both male and female Tg26 mice showed similar short-term spatial memory deficits (as indicated by the exploratory preference in the one-hour retention probe test compared to WT mice), the male Tg26 group performed much worse (*p* < 0.001) than the female Tg26 group when compared with the WT mice (Figure 2C). While the exploratory preference of the male WT mice was more than 60.50 ± 4.3%, it was only about 18.4 ± 6.2% in the Tg26 mice (Figure 2C, left); however, only a small non-significant difference was observed in the female groups (Figure 2C, right). These results indicate that Tg26 mice, especially male mice, have impaired spatial memory and discrimination compared to WT mice.

#### 3.1.3. Test of Spatial Learning and Memory

We used the BM test to evaluate spatial learning and memory in Tg26 and WT mice. The test was conducted on a circular open arena with 20 escape holes located around the periphery of the platform. Bright overhead lighting was used to create an aversive stimulus, encouraging the mice to escape from a brightly lit, exposed circular open arena to a small dark escape box located under one of several holes at the periphery of the arena.

**Figure 3:**
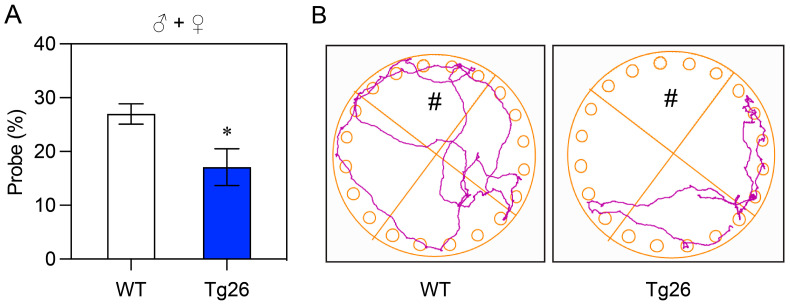
Evaluation of spatial learning and memory in Tg26 and WT mice using the BM test. (**A**) Comparison of the time spent in the escape quadrant by Tg26 (*n* = 9) and WT (*n* = 14) mice at the age of 12–14 weeks old, presented as the percentage of probe time for the escape quadrant. Mean ± SEM are shown as bar graphs, with statistical significance indicated as * (*p* < 0.05). (**B**) Representative paths of WT and Tg26 mice during the probing process. The symbol # represents the correct quadrant where the escape can be found. The colored lines are the representative paths mice took during the exploration process.

After a four-day acquisition training period (day 1–4), the escape tunnel was removed and replaced with a shallow tray similar to the other holes. On day 5, the animals were allowed to explore the Barnes Maze platform, and the time spent in the correct quadrant (where the escape zone used to be) was measured. As shown in Figure 3A, Tg26 mice (*n* = 9) spent significantly (*p* < 0.05) less time in the correct quadrant compared to the WT mice (*n* = 14). Consistent with this observation, the path that Tg26 mice took during the exploration process was also much more limited compared to the WT mice (Figure 3B).

### 3.2. Transgenic HIV-1 Infection in the Cortex and Hippocampus of Tg26 Mice Upregulates SUR1-TRPM4 Related Neuroinflammation in Reactive Astrocytes and Downregulates Neuronal Synaptic Regulation

In our previous study, we demonstrated that transgenic expression of the HIV-1 gene in Tg26 mice induces a proinflammatory response in astrocytes mediated by SUR1-TRPM4 channels [7]. In this study, we aimed to investigate whether this neuroinflammatory response is correlated with impaired neurobehavioral and locomotor abilities and reduced neuronal synaptic functions that we observed. To do so, we used IHC and qRT-PCR to detect possible changes in gene expression and protein production in the cortex and hippocampus of WT and Tg26 mice of mixed sexes. Astrocytic and neuronal functions were examined in the cortex and hippocampus because HIV can be found in various regions of the brain, including hippocampus and prefrontal cortex among others [7,44]. HIV primarily infects the immune cells in the brain, such as astrocytes in microglia, which represent the most abundant type of glial cells. Astrocytes are important supportive cells in the brain, and disruption of their normal functions can lead to inflammation and damage to neurons and other brain cells [7,44], which can ultimately result in cognitive and neurological impairments, collectively known as HAND [8,45].

#### 3.2.1. Immunohistochemistry Analysis 

**Figure 4:**
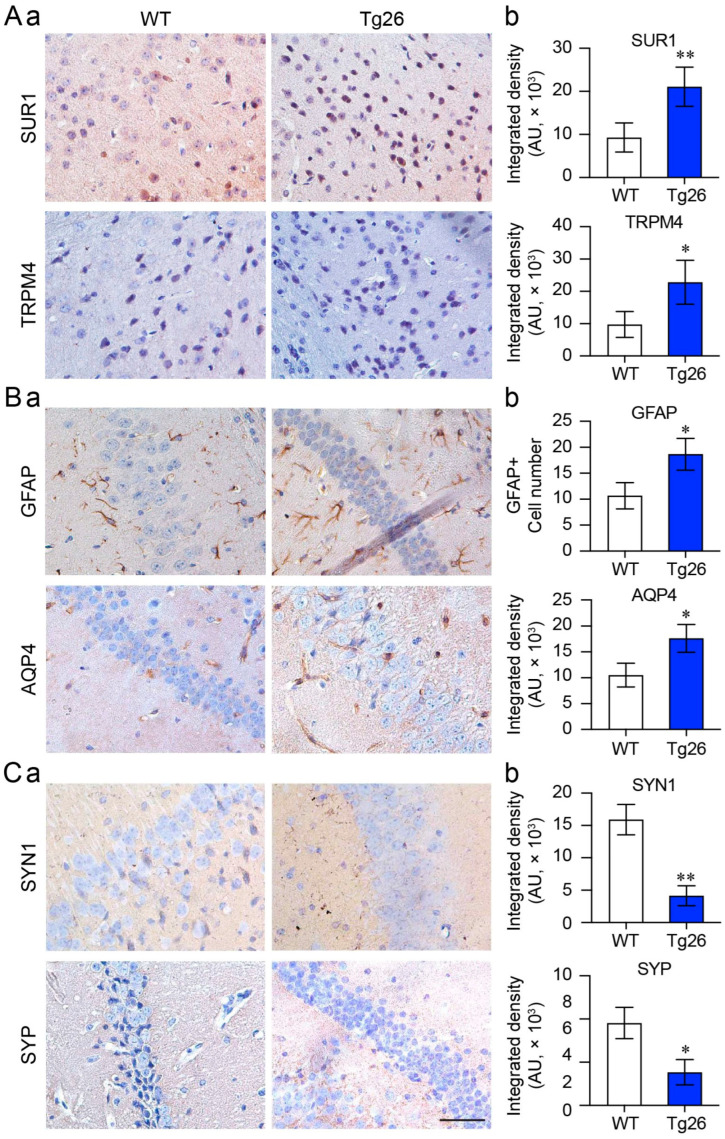
Comparison of proinflammatory and synaptic protein expression in the cortex and hippocampus of WT and Tg26 mice. The figure shows representative IHC images and quantification of the SUR1-TRPM4 channel (**A**), astrocyte activation (GFAP and AQP4) (**B**), and synapses (SYN1 and SYP) (**C**) in the hippocampus brain tissue of WT and Tg26 mice at 16 weeks of age with mixed sexes. (**a**) Immunohistochemistry microscopic images, (**b**) graphic representation of (**a**). The data are presented as Mean ± SD. An unpaired *t*-test was used to determine statistical significance between the two testing groups, with * *p* < 0.05 and ** *p* < 0.01 indicating significance. Scale bars are 100 µm.

First, we conducted IHC analysis to detect possible changes in protein expression between WT and Tg26 mice. We observed a significant increase in the expression of both SUR1 and TRPM4 in Tg26 mice compared with WT mice (Figure 4A, *p* < 0.01). Consistent with our previous findings, we also observed an increase in GFAP expression, indicating astrocyte activation, and upregulation of AQP4 (Figure 4B), a water channel predominantly expressed by astrocytes in the CNS. Additionally, we evaluated two major synaptic regulators, SYN1 and SYP, and found that their expression was significantly decreased in Tg26 mice (Figure 4C). Note that Western blot analysis generally offers advantages over immunostaining for protein quantification. However, considering that HIV-1 primarily infects specific regions of the brain, such as the cortex and hippocampus, where the expression of inflammatory and synaptic proteins may be further localized within these infected regions, we decided to carry out immunostaining of a specific protein of interest in a targeted region that we believe could provide a more accurate representation than protein quantification of a mixed tissue sample.

#### 3.2.2. Analysis by Quantitative Reverse Transcription PCR

**Figure 5:**
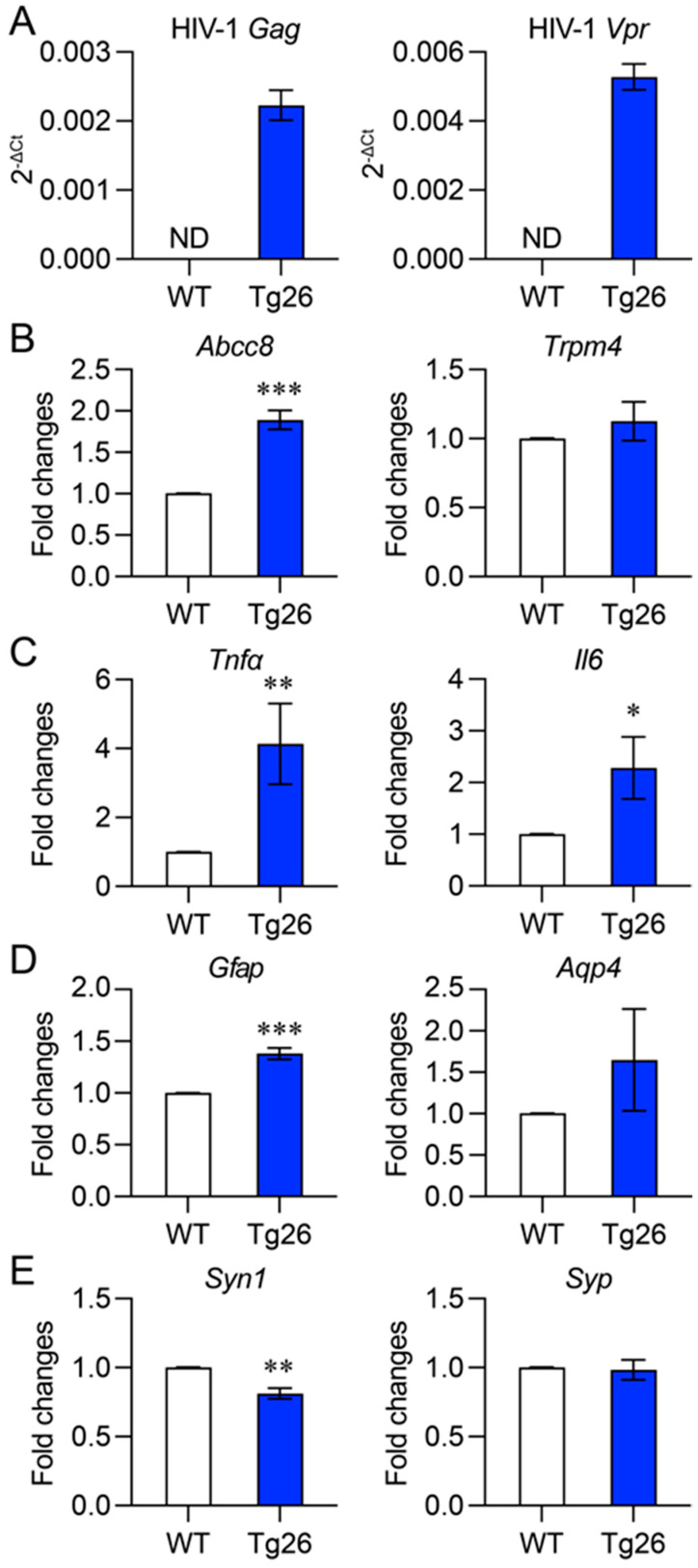
Comparison of proinflammatory and synaptic gene transcription in the cortex and hippocampus of WT and Tg26 mice. (**A**) Verification of transgenic HIV-1 gene expression in the Tg26 and WT mice of mixed sexes was performed by qPCR. Representative transcriptional gene expression of proinflammatory markers *Abcc8* (which encodes SUR1 protein) and *Trpm4* (**B**), *Tnfα* and *Il6* (**C**) astrocyte activation (*Gfap* and *Aqp4*) (**D**), and neuronal synaptic function markers *Syn1* and *Syp* (**E**) were measured in the cortex and hippocampus brain tissue of WT and Tg26 mice. The gene expression profiles are presented as relative ratio to house-keeping *Gapdh* gene (as indicated by 2^−ΔCT^ value) in each sample (**A**) or relative fold change to WT (**B**–**E**). *Gapdh* gene was used as an internal control. The data are presented as Mean ± SD. An unpaired *t*-test was used to determine statistical significance between the two testing groups, with * *p* < 0.05, ** *p* < 0.01, and *** *p* < 0.001 indicating the levels of significance.

We also compared gene expression levels in the cortex and hippocampus of Tg26 and WT mice using qRT-PCR analysis. We verified transgenic HIV-1 gene expression in Tg26 mice by detecting the Gag polyprotein and HIV-1 Vpr, which were previously shown to contribute to SUR1-mediated neuroinflammation and neurotoxicity [7] (Figure 5A). Consistent with our IHC results (Figure 4), we found that the transcriptional activity of the SUR1-encoding gene (*Abcc8*) was significantly elevated in Tg26 mice compared with WT mice (Figure 5B). Similarly, gene transcription of the reactive astrocytic markers *Gfap* and *Aqp4* was also increased in Tg26 mice compared with WT mice (Figure 5C,D). In contrast, the expression level of synaptic regulator *Syn1* was decreased in Tg26 mice, consistent with reduction observation in the IHC results. However, we found some discrepancies between the IHC and qRT-PCR results. Although qRT-PCR showed higher gene transcription levels of *Aqp4* in Tg26 mice than in WT mice, the difference was not statistically significant (Figure 5C, right). Additionally, there was little or no difference between Tg26 and WT mice in the gene expression profiles of *Trpm4* (Figure 5B, right) or *Syp* (Figure 5D, right).

Overall, our results suggest that the differences observed at the protein level by IHC analysis, and the differences measured at the gene transcriptional level by qRT-PCR are largely concordant. The observed discrepancies could potentially be due to heterogenicity of the mouse tissues used in two different assays. This indicates that transgenic HIV-1 infection in Tg26 mice leads to upregulation of SUR1-TRPM4-mediated reactive astrocytosis and downregulation of synaptic protein production in the cortex and hippocampus, potentially contributing to the observed impaired neurobehavioral and locomotor abilities.

## 4. Discussion

Despite our success in using ARTs to eliminate viral replication in the bloodstream, a significant challenge remains due to poor penetration of certain antiviral drugs into the CNS. This limitation results in the virus persistently replicating and causing chronic inflammation in the brain, leading to HAND [1,2]. Therefore, gaining a better understanding of the relationship between HIV-1 infection and neuroinflammation in the brain is crucial in developing more effective therapeutic strategies to combat HAND.

This study builds upon our previous findings that HIV-1 infection and Vpr trigger activation of the SUR1-TRPM4 channel, inducing neuroinflammation through NF-kB-mediated cytokine activation, and likely contributing to HAND [7]. Here, we further investigated the relationship between SUR1-TRPM4-mediated neuroinflammation and cognitive deficits using an HIV transgenic Tg26 mouse model [17]. The Tg26 mouse model was used because it has been shown that the impact of HIV-1 infection on neurobehavioral impairment and neuroinflammation in the brain of Tg26 resembles the abnormalities found in HAND [17,18,23]. We compared the cognitive and memory abilities of Tg26 mice to those of WT mice using various tests, including locomotor activity tests, recognition memory tests, and spatial learning and memory tests. Our results demonstrated that Tg26 mice have impaired cognitive skills and reduced learning abilities, particularly in spatial learning and memory, compared to WT mice. We then analyzed the correlation between HIV-induced neuroinflammation and synaptopathy, as well as impaired learning and memory, using IHC and qRT-PCR analyses. Our results suggest a high degree of concordance between SUR1-TRPM4-induced neuroinflammation in reactive astrocytes and synaptopathy, which may be reflected by impaired spatial learning and memory.

The effect of HIV-1 infection on impairment of cognitive learning and memory has been well documented in HIV-1 infected individuals [8,45], as well as in various animal models [21,22,23]. Consistent with those earlier findings, our study also demonstrated that transgenic HIV-1 infection in Tg26 mice leads to a decline in locomotor activity and recognition memory when compared to WT mice (Figure 1). Furthermore, their spatial memory and discrimination abilities are compromised as well (Figure 2). This was further confirmed by the results of the Barnes Maze tests, where Tg26 mice spent significantly less time (*p* < 0.05) in the correct quadrant and their movement was noticeably more restricted compared to the WT mice (Figure 3). Collectively, these observations strongly suggest a notable impairment in learning and memory in Tg26 mice compared to the WT mice. While there was no clear sexual difference in overall cognitive outcomes in the Tg26 mouse model of HANDS, our study did find significant differences in spatial memory and discrimination abilities in male mice (Figure 2C, left). This is in line with other studies that have demonstrated sex-dependent differences in cognitive behaviors and memory in animal models of various neurological conditions, including HIV-1 infection. For example, Moidunny et al. [46] associated sex-dependent contextual fear memory deficits in male Tg26 mice with reduced SYN1, while Koss et al. [47] found that female mice performed better in a working memory task, but worse in a reference memory task compared to male mice. Thus, while the impact of sex on cognitive outcomes in HAND may be more complex, our findings add to a growing body of literature that highlights the importance of considering sex differences in research on cognitive function in neurological disorders.

In our previous studies, it was established that HIV-1 infection and Vpr trigger neuroinflammation through the activation of the SUR1-TRPM4 channel, which is associated with NF-kB-mediated cytokine production and HAND [7]. In this study, by using the Tg26 mouse model of HAND [17], we found a strong correlation between this neuroinflammation in the reactive astrocytes of cortex and hippocampus and deficiencies in neuronal synaptic regulation (Figure 4 and Figure 5). GFAP and AQP4 were used here because they are important biomarkers for neuroinflammation and are associated with reactive astrocytosis [35]. Astrocytes are the most abundant cell type within the CNS, which are crucial for maintaining CNS homeostasis and managing neuronal pathology [48]. They are also the target cells for immune mediators and HIV-1-associated neurotoxicity that led to reactive astrogliosis, a common characteristic of HAND.

Synaptic degeneration, which is also associated with HAND, is regulated by astrocytes’ dynamic interaction with neurons to control synaptic transmission. Here, we used two well-established markers, SYN1 and SYP, to monitor neuronal synapses due to their roles in synaptic vesicle cycling and synaptic function [49]. Studies have shown that SYN1 and SYP expression is altered in brain regions involved in learning and memory in animal models of Alzheimer’s disease (AD) and other neurodegenerative disorders, suggesting that synaptic dysfunction may contribute to cognitive impairment in these conditions [36]. Other studies have used SYN1 and SYP as markers to investigate aging-related synaptic plasticity and cognitive function [50].

Consistent with our early results, SUR1-TRPM4 channels were found to be highly elevated in Tg26 mice along with GFAP and AQP4 elevations, indicating reactive astrocytosis and neuroinflammation. Corresponding reductions in neurosynaptic responses, as indicated by the downregulation of SYN1 and SYP, suggested synaptopathy as a possible mechanism underlying cognitive and motor skill deficits. These findings together suggest that the ionic imbalance caused by the upregulation of the SUR1-TRPM4 channel in reactive astroctyes might be associated with synaptic degeneration and cognitive deficit. Therefore, our findings may establish a role of astrocyte-mediated synaptic plasticity dysfunction in cognitive deficit and suggest that astroglia networks control synaptic strength by modulating extracellular homeostasis.

The correlation we observed does not establish a causal relationship between SUR1-TRPM4-mediated neuroinflammation and cognitive and memory changes. Further studies are necessary to confirm these findings. Additionally, while we found that HIV-1 Vpr triggers elevation of the SUR1-TRPM4 channel leading to neuroinflammation and neurotoxicity in astrocytes [7], we have no evidence to suggest that Vpr also contributes to the observed cognitive and memory changes. Further research is needed to explore this possibility.

In summary, this study suggests a possible relationship between SUR1-TRPM4-mediated neuroinflammation and synaptopathy with impairments of learning and memory in mice with HANDS. These findings may shed light on the functional relationship between SUR1-mediated neuroinflammation and HAND, which could potentially help us to develop new therapeutic strategies for individuals living with HIV.

## Data Availability

Not applicable.

## Supplementary Materials

The following supporting information can be downloaded at: https://www.mdpi.com/article/10.3390/jcm12165169/s1, Table S1: List of primary antibodies; Table S2: Primer sequences used in this study.
