# Supplementary material for: Correlation of HIV-Induced Neuroinflammation and Synaptopathy with Impairment of Learning and Memory in Mice with HAND
Source: J Clin Med. 2023 Aug 8;12(16):5169. doi: 10.3390/jcm12165169 (PMC10455390; doi:10.3390/jcm12165169)
Supplement: Supplementary file 1 [file jcm-12-05169-s001.zip › jcm-2418699-supplementary.pdf]

Supplemental Materials

Table S1. List of primary antibodies.

**Antibody**	**Target**	**Vendor / Cat #**	**Dilution**	**Species & Clonality**
Anti-SUR1	Sulfonylurea Receptor- 1	Custom made [25]	1:250	Rabbit Polyclonal
Anti-TRPM4	Transient Receptor Potential Channel Subfamily M Member 4	Millipore Sigma / ABN418	1:200	Rabbit Polyclonal
Anti-GFAP	Glial Fibrillary Acidic Protein	Millipore Sigma / AB5541	1:100	Chicken Polyclonal
Anti-AQP4	Aquaporin 4 Water Channel	Santa Cruz Biotechnology / SC-32739	1:100	Mouse Monoclonal
Anti-Synapsin-1	Synapsin-1 / synapse marker	Biosensis / R-1829	1:200	Rabbit Polyclonal
Anti-Synaptophysin	Synaptophysin / synapse marker	Novus Biologicals / NB300-653	1:200	Rabbit Polyclonal

Table S2. Primer sequences used in this study.

**Gene**	**Species**	**Forward primer**	**Reverse primer**
Gag	HIV-1	CATGTTTTCAGCATTATCAGAAGGA	TGCTTGATGTCCCCCCACT
Vpr	HIV-1	GATACTTGGGCAGGAGTGGA	TGGCTCCATTTCTTGCTCTC
Tnfα	Mouse	ATGAGCACAGAAAGCATGA	AGTAGACAGAAGAGCGTGGT
Il6	Mouse	CCACTTCACAAGTCGGAGGC	TCTGCAAGTGCATCATCGTTGT
Abcc8	Mouse	GCCAGCTCTTTGAGCATTGG	AGGCCCTGAGACGGTTCTG
Trpm4	Mouse	TGTTGCTCAACCTGCTCATC	GCTGTGCCTTCCAGTAGAGG
Gfap	Mouse	GAGAACAACCTGGCTGCGTAT	TGCCTCGTATTGAGTGCGAAT
Aqp4	Mouse	GCATCGCTAAGTCCGTCTTCTAC	GTGAACACCAACTGGAAAGTGATTA
Syn1	Mouse	GCTCAGCAGCACAACATACCC	TGCCCAATCTTCTGGACACG
Syp	Mouse	TGCCAACAAGACGGAGAGTG	CCGAGGAGGAGTAGTCACCAAC

Note: The primer sequences targeting HIV-1 *Gag*, *Vpr*, *Tnfα*, *Abcc8*, and *Trpm4* were described previously [7].
